# From Conception to Development: Investigating PROTACs Features for Improved Cell Permeability and Successful Protein Degradation

**DOI:** 10.3389/fchem.2021.672267

**Published:** 2021-04-20

**Authors:** Carlotta Cecchini, Sara Pannilunghi, Sébastien Tardy, Leonardo Scapozza

**Affiliations:** ^1^School of Pharmaceutical Sciences, University of Geneva, Geneva, Switzerland; ^2^Pharmaceutical Biochemistry/Chemistry, Institute of Pharmaceutical Sciences of Western Switzerland, University of Geneva, Geneva, Switzerland

**Keywords:** proteolysis targeting chimeras, ubiquitin-proteasome system, drug discovery, cell permeability, protein degradation, PROTAC technology

## Abstract

Proteolysis Targeting Chimeras (PROTACs) are heterobifunctional degraders that specifically eliminate targeted proteins by hijacking the ubiquitin-proteasome system (UPS). This modality has emerged as an orthogonal approach to the use of small-molecule inhibitors for knocking down classic targets and disease-related proteins classified, until now, as “undruggable.” In early 2019, the first targeted protein degraders reached the clinic, drawing attention to PROTACs as one of the most appealing technology in the drug discovery landscape. Despite these promising results, PROTACs are often affected by poor cellular permeability due to their high molecular weight (MW) and large exposed polar surface area (PSA). Herein, we report a comprehensive record of PROTAC design, pharmacology and thermodynamic challenges and solutions, as well as some of the available strategies to enhance cellular uptake, including suggestions of promising biological tools for the *in vitro* evaluation of PROTACs permeability toward successful protein degradation.

## Introduction

The use of small-molecules for target modulation represents a classical approach in drug discovery, with a proven track record up to clinical use on a plethora of biological targets. Historically, the pharmacological intervention focused on targets with well-defined active sites suitable for the accommodation of a small molecule. Nevertheless, a wide variety of proteins do not completely fulfill this characteristic and are considered “undruggable,” thereby representing challenging targets. Alternative approaches, such as monoclonal antibodies, have tried to overcome this drawback, but their operating range is limited to extracellular or cell surface targets (Lazo and Sharlow, 2016).

Additionally, other strategies acting at the genetic level can be used to knockdown intracellular proteins, including scaffolding proteins, transcription factors, and non-enzymatic proteins (Valeur et al., 2017). Nucleic acid-based agents, such as antisense oligonucleotides (ASOs) or small interfering RNAs (siRNAs) have successfully provided high efficacy in preclinical studies and some of them have been approved for clinical trials. In parallel, the recent advent of CRISPR–Cas9 technology offers the possibility of *in situ* genome editing to achieve gene knockout (Doudna and Charpentier, 2014). However, despite their therapeutic potential, these strategies present hurdles in terms of cellular delivery, stability, biodistribution, and selectivity (Deng et al., 2014).

**Figure 1:**
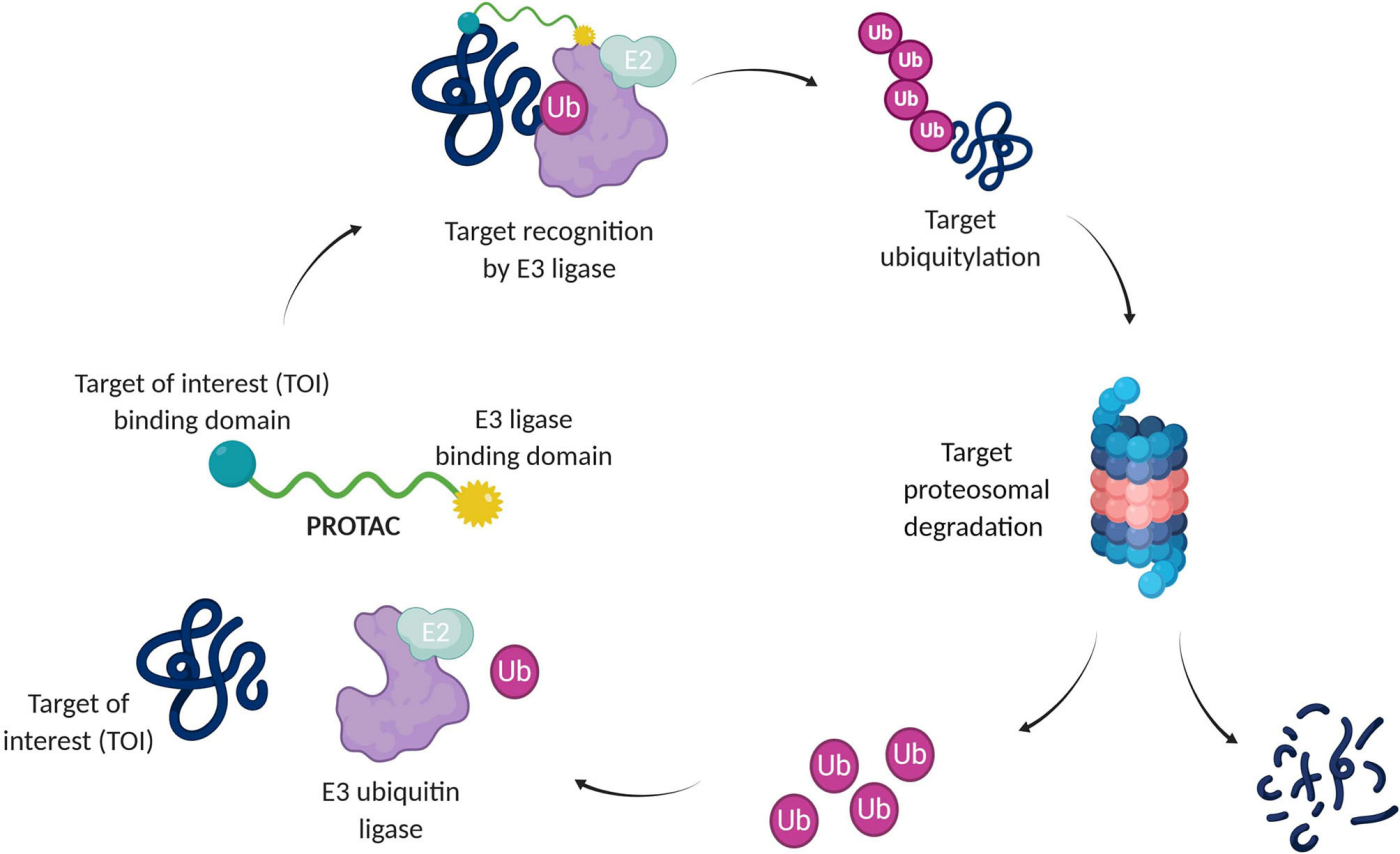
Targeted protein degradation by PROTACs. A PROTAC simultaneously binds a target of interest (TOI) and an E3 ubiquitin ligase complex, leading to ubiquitination and degradation of the TOI via the UPS. E2, Ubiquitin-conjugating enzyme; Ub, Ubiquitin.

A recent pharmacological strategy to circumvent the target's lack of “druggability” employs chimeric molecules called Proteolysis Targeting Chimeras (PROTACs). Recent years have shown exciting advances in PROTAC technology, which has emerged as a potent tool to rapidly and reversibly deplete endogenous proteins involved in many diseases (Cromm and Crews, 2017; Lai and Crews, 2017; Churcher, 2018; Pettersson and Crews, 2019). PROTACs are heterobifunctional molecules able to specifically bind a target protein and induce its degradation by specifically recruiting a given E3 ubiquitin ligase (Bondeson et al., 2015, 2018). Contrary to protein inhibition, this technology benefits from the cell's own protein degradation pathway -Ubiquitin-Proteasome System (UPS)-to specifically remove labeled proteins (Nandi et al., 2006) (Figure 1).

The UPS consists of a multiple enzymatic step process with sequential action of ubiquitin-activating enzymes (E1), ubiquitin-conjugating enzymes (E2), and ubiquitin ligases (E3). The addition of polyubiquitin chains to a target protein serves as a recognition marker for its proteolytic degradation through the proteasome (Nandi et al., 2006).

By taking advantage of this machinery, PROTACs can drive the knockdown of a large panel of proteins, including nuclear receptors, transcription factors, skeleton proteins, enzymes, and regulatory proteins, which are dysregulated in cancer and other diseases (Sun et al., 2019).

## Protein Degradation vs. Protein Inhibition

### PROTACs act Through Event-Driven Pharmacology

**Figure 2:**
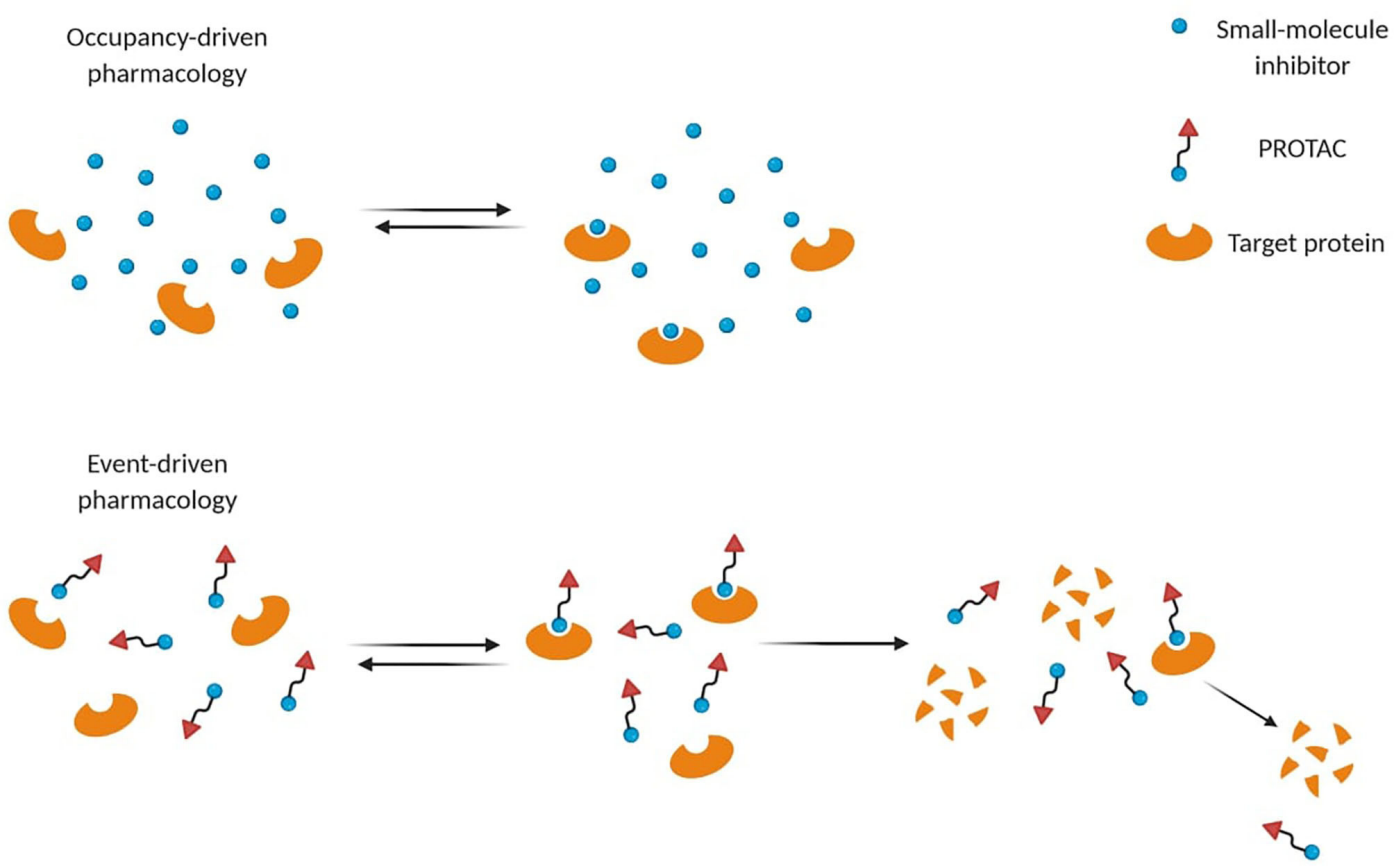
Different mode-of-action of small-molecule inhibitors and PROTACs. Small-molecule inhibitors often require higher concentrations to be effective, while PROTACs act through event-driven pharmacology that leads to targeted protein degradation at lower concentrations.

Small-molecule inhibitors act through occupancy-driven pharmacology, which blocks malfunctioning proteins via inhibition, while PROTACs operate through event-driven pharmacology, meaning that protein function is controlled by decreasing the cellular protein level (Figure 2) (Cromm and Crews, 2017). Through a transient and reversible association between a PROTAC and its substrate, one single molecule can induce degradation of more proteins sequentially, leading to a sub-stoichiometric protein knockdown. Several advantages arose from PROTAC's engagement over occupancy-based inhibitors. Thanks to their mechanism of action (MoA), a plethora of PROTACs showed efficacy at nM concentrations to achieve high degradation potency (Cromm and Crews, 2017). Moreover, they ensure longer time efficacy than small-molecule inhibitors, since the restoration of protein function requires protein resynthesis (Lai and Crews, 2017). Therefore, the need for high equilibrium target occupancy or long drug exposure in the diseased tissue may be reduced.

Unlike small-molecule inhibitors, these chemical probes do not require a catalytic binding site on the target since they can induce its degradation by binding alternative non-catalytic sites (Lai and Crews, 2017). Indeed, even targets defined “undruggable” by occupancy-based ligands can be detected and degraded (Lai and Crews, 2017; Cromm et al., 2018). In cancer, mutations close to the inhibitor binding pocket cause drug resistance by making inhibition less effective or ineffective (Buhimschi et al., 2018). PROTACs may circumvent this phenomenon due to their ability to form transient interactions to induce functional knockdown of their targets (Buhimschi et al., 2018). For instance, a potent androgen receptor (AR) degrader (ARCC-4) resulted better at overcoming resistance in different cell-based models compared to its parent inhibitor (Salami et al., 2018). Nevertheless, other resistance mechanisms such as genomic alterations in the ubiquitin-proteasome system could also affect PROTACs efficacy (Ottis et al., 2019; Zhang et al., 2019a).

### Enhanced Target Selectivity With PROTACs

Recent studies reported that non-selective inhibitors could be converted into selective PROTACs (Zengerle et al., 2015; Gadd et al., 2017; Yang et al., 2018; Khan et al., 2019). Among bromodomain and extra terminal (BET) proteins (BRD2, BRD3, BRD4), BRD4 has been strongly linked to cancer and inflammatory diseases, thereby representing an attractive target (Zengerle et al., 2015). Conjugating a pan-BET inhibitor JQ1 to a VHL ligand provided a PROTAC (MZ1) that selectively degraded BRD4 over BRD2 and BRD3 (Zengerle et al., 2015). Likewise, VHL- and CRBN- based PROTACs targeting tumor-related histone deacetylase 6 (HDAC6) derived from a pan-HDAC inhibitor showed potent and selective degradation of HDAC6 (Yang et al., 2018, 2020). These findings suggest that PROTACs can exceed the binding selectivity of the TOI ligand by adding a layer of specificity, which probably relies on the choice of the recruited E3 ligase (Bondeson et al., 2015; Winter et al., 2015; Lai et al., 2016). For example, Crews et al. demonstrated that only CRBN-based PROTACs were capable of degrading oncoprotein BCR/Abl and c-Abl, whereas VHL-based PROTACs only degraded c-Abl (Lai et al., 2016). Although most of the mechanistic insights behind these findings are still to be clarified, the enhanced selectivity headed by PROTACs has proven to address issues encountered with small-molecule inhibitors, such as reducing specific off-target effects. A selective BCL-X_L_ degrader (DT2216) demonstrated *in vivo* activity without producing thrombocytopenia, a significant side effect previously shown by BCL-X_L_ inhibitors (Khan et al., 2019). These promising studies raise hope that non-selective small-molecule inhibitors may be exploited and engineered into selective and efficient PROTACs (Bondeson et al., 2015; Lu et al., 2015; Burslem et al., 2018).

## Aspects of Protac Design

From a structural point of view, PROTAC design relies on the combination of three different chemical moieties: an E3 ligase binder, a target of interest (TOI) ligand, and a linker, which connects the two parts (Figure 1). Finding the best combination for these three elements requires an attentive study of the structural characteristics of the E3 ligase and the TOI complemented by molecular modeling and dynamics (Westermaier et al., 2015; Cecchini et al., 2020). In particular, the spatial orientation and the alignment of both elements allow the formation of a ternary complex and are crucial for an effective ubiquitin-protein knockdown. Moreover, the stability of the latter, the rate at which the labeled target is processed by the proteasome, and the rates of *de novo* protein synthesis affect PROTAC potency (Bondeson et al., 2015; Churcher, 2018).

### TOI Ligand Structure-Activity Relationship (SAR) Is the Minimal Requirement to Rationally Generate New PROTACs

The crystal structure of the TOI ligand bound to its target or its structure-activity relationship (SAR) information is often required to guide the design of new protein degraders. Based on this, it is possible to optimize such molecules by identifying linker attachment points in regions that are not involved in the target binding. However, the lack of a well-characterized ligand capable of interacting with the desired target limits early-stage drug discovery projects, involving novel biological targets with poorly understood pharmacology (Chessum et al., 2018; Bai et al., 2019).

Additionally, a non-solvent-exposed pocket that fully embeds its ligand represents a tremendous challenge for finding a suitable attachment point for the linker. For research purposes and target validation, orthogonal approaches such as HaloPROTAC (Buckley et al., 2015; Tovell et al., 2019), or dTAG (Nabet et al., 2018), have been developed. In both cases, with the intent to circumvent the time-consuming process required to develop a new PROTAC, the TOI is fused to an exogenous tag that is targeted by its specific PROTAC. Nevertheless, these tag-based systems abrogate some of the advantages of PROTAC technology concerning portability and no requirement for genetic manipulation since tags are introduced into the endogenous locus via gene editing.

To foster the acquisition of the structural information needed for facilitating PROTAC development, X-ray crystallography and, in recent years, cryo-electron microscopy (cryo-EM) (Yip et al., 2020) have become the favorite methods. In addition to these experimental methods, a new AI technology called AlphaFold (Senior et al., 2019, 2020) recently emerged as a tool to accurately predict a protein's 3D shape from its amino-acid sequence. This promising technology is seen as having the potential of accelerating structures' resolution by enabling insights into the function and malfunction of proteins and fostering the discovery of drugs, including PROTACs having the appropriate physico-chemical properties for cell penetration.

### Challenges in Expanding the E3 Ligase Toolbox in Targeted Protein Degradation

The choice of E3 ligase determines the success of protein knockdown. On a given target, distinct degradation profiles are observed depending on the recruited E3 ligase (Steinebach et al., 2020). Emerging evidence suggests that E3 ligases with tissue-selective expression profiles are expected to present unique opportunities for therapeutic applications, even if their mechanisms have not yet been comprehensively studied (Schapira et al., 2019). So far, only a few E3 ligases, including Cereblon (CRBN), Von Hippel–Lindau (VHL), Mouse double minute 2 homolog (MDM2), and Cellular Inhibitor of Apoptosis Protein 1 (cIAP1), have been targeted by PROTACs (Schapira et al., 2019), while more than 600 E3 ligases are predicted to be encoded by the human genome. Therefore, it is highly desirable to extend the panorama of E3 ligase to be used in the context of targeted protein degradation. While the traditional approach is based on recruiters that reversibly bind to their corresponding E3 ligases, it has been demonstrated that targeting E3 ligases with covalent reactive small-molecules can also be used as E3 ligase recruiting strategy. In this perspective, chemical proteomics represents a powerful tool for large-scale profiling of protein-small molecule interactions across the native proteome (Zhou and Xiao, 2020). A library of electrophilic covalent fragments (called “scouts”) targeting several “undruggable” proteins was built in Cravatt's lab to help discover electrophilic E3 ligands in cell-lysates (Backus et al., 2016; Zhang et al., 2019b). In this context, a chloroacetamide-bearing PROTAC (KB02-SLF) was used as bait to alkylate previously uncharacterized E3 ligases, such as DCAF16, thereby providing a new valid candidate for PROTAC technology. Similarly, recent studies showed the discovery of new “ligandable” sites on RNF4 (Ward et al., 2019) and RNF114 ligases (Spradlin et al., 2019). The success of these E3 covalent highly reactive recruiters fostered the research toward covalent but reversible E3 ligase interactions, as shown for bardoxolone recruiting KEAP1 ligase (Tong et al., 2020).

Alternatively, the use of molecular glues, whose mode of action is different from PROTACs, may represent a further tool to expand the panorama of recruiting E3 ligases. Their mechanism relies on novel protein-protein interactions between a substrate receptor of an E3 ubiquitin ligase and a target protein to induce its degradation (den Besten and Lipford, 2020). Such strategies show the potential to increase the repertoire of small-molecule ligand–E3 ligase pairs in the PROTAC toolbox. However, how to evaluate the ability of E3 ligase components to hijack neo-substrate degradation remains an important challenge. In this regard, Pinch et al. conveyed a method so-called Covalent Functionalization Followed by E3 Electroporation into live cells (COFFEE) that bypasses the need for hit finding to identify specific E3 ligase binders (Pinch et al., 2020). In this work, they covalently linked a BRD4 ligand, JQ1, and the multi-kinase inhibitor, dasatinib, to VHL and other E3 ligases via their solvent-exposed cysteines and they further electroporated the recombinant E3 ligases into live cells to form functional E3 ubiquitin ligase complexes capable of TOI degradation. This strategy represents an orthogonal approach to chemical proteomics and the use of molecular glues to expand the E3 toolbox in targeted protein degradation against neo-substrates. In terms of chemistry, this expansion may lead to a broader chemical space for putative ligase binders and, consequently, an improvement of the PROTACs physico-chemical properties influencing cellular uptake.

### The Chemistry of the Linker Is Instrumental for PROTAC Success

PROTAC requires both an E3 ligase-recruiting moiety and a TOI ligand, each possessing a suitable solvent-exposed position to connect the parts through a linker. The linker geometry and chemical composition should orient the E3 ligase and the TOI in such a way as to put the two proteins in proximity and allow favorable protein-protein interactions (PPIs) to occur (Bondeson et al., 2018; Nowak et al., 2018). In this way, the transfer of ubiquitin units within the ternary complex can lead to potent target degradation through the ubiquitin-proteasome system. Polyethylene glycol (PEG) chains are the most common type of linkers used so far due to their polarity and flexible nature (Bondeson et al., 2018; Pettersson and Crews, 2019; Sun et al., 2019), but other examples include pure lipophilic alkyl chains (Winter et al., 2015; Shah et al., 2020; Zhang et al., 2020a; Zhou et al., 2020). In some cases, rigidifying the linker through heterocyclic scaffolds (e.g., piperazine/piperidines) led to a stable ternary complex formation and potent protein degradation (Farnaby et al., 2019; Han et al., 2019). This might be explained by a reduced loss of the system's entropy. Moreover, the introduction of polar pyridine/piperidine motif can modulate the PROTAC physico-chemical properties in such a way as to improve aqueous solubility and cell permeability.

One of the most common strategies to combine TOI and ligase binder is the use of click chemistry (Wurz et al., 2018), a copper-catalyzed reaction that consists of a Huisgen 1,3-dipolar cycloaddition, which involves an azide and an alkyne moiety. Besides the wide applicability and high compatibility of this linking strategy, the resulting triazole ring may also represent a metabolic advantage compared to linear linkers, which are more easily exposed to oxidative metabolism *in vivo* (Xia et al., 2019).

The linker length plays a crucial role in defining the linker geometry. To elucidate this role, Crew et al. generated a panel of VHL-based PROTACs targeting serine/threonine kinase TANK-binding kinase 1 (TBK1), a protein implicated in cancer (Crew et al., 2018). Interestingly, they found that PROTACs with linkers of <12 atoms demonstrated no appreciable degradation activity. In line with these findings, VHL-based PROTACs targeting estrogen receptor (ER)-α showed to promote greater degradation with an optimal linker length of 16 atoms (Cyrus et al., 2011). These observations suggest that a minimum linker length is required to allow the E3 ligase and the target to come together without incurring steric conflicts.

The linker itself may also significantly impact PROTAC's selectivity. For example, Crews' group observed that a small increase in linker length changed the degradation profile from a PROTAC able to target both epidermal growth factor receptor (EGFR) and human epidermal growth factor receptor 2 (HER2) into one that selectively degraded only EGFR (Burslem et al., 2018). Conversely, in a study conducted by Nowak et al. (2018), it has been found that shorter linkers exhibit higher selectivity because the number of possible conformations is reduced.

Such results underline the importance of the linker length in geometry to provide different selectivity profiles in PROTAC-based protein degradation and suggest that degradation and selectivity can be modulated in a sophisticated manner (Smith et al., 2019). Unfortunately, a clear understanding of the so-called “linkerology” is missing. Nevertheless, the advent of new molecular modeling tools to study the ternary complex, such as PRosettaC (Zaidman et al., 2020; Bai et al., 2021), may address the issues related to the linker choice and accelerate PROTAC development.

## Elements of Thermodynamics

### What Is Needed for High Degradation Potency?

Small-molecule inhibitors' efficacy is mainly dependent on their binding affinity for the target, while PROTAC degradation potency can also be influenced by other factors. This has been shown in a paper reporting that PROTACs derived from a potent BET inhibitor exhibited lower efficacy than the original high-affinity inhibitor (Chan et al., 2018). Furthermore, Crews et al. introduced the concept of a productive ternary complex (TOI-PROTAC-E3 ligase) formation stabilized by PPIs. By developing CRBN- and VHL- based PROTACs from a well-known kinase inhibitor, potentially capable of degrading more than 100 kinases, they observed that PROTACs incorporating low-affinity TOI recruiting moieties displayed potent degradation capacity since they could form a robust ternary complex (Bondeson et al., 2018).

The first element to answer the question is that the high binding affinity of the TOI ligand is a valuable starting point and ensuring the formation of a stable ternary complex between the TOI, the PROTAC, and the recruited E3 ligase seems important but strongly debated.

### Ternary Complex Formation and Cooperativity

Several studies analyzed the ternary complex formation thermodynamics (Gadd et al., 2017; Hughes and Ciulli, 2017; Chan et al., 2018; Nowak et al., 2018; Zorba et al., 2018; Testa et al., 2020). Pioneer in this field, Ciulli's group recently solved the first crystal structure of a ternary complex (BRD4-MZ1-VHL) at 2.7Å resolution (PDB 5T35) (Gadd et al., 2017). They illustrated how induced electrostatic surface interactions between the target and the E3 ligase in the presence of the PROTAC play a central role in the stability of the ternary complex. Indeed, favorable interactions between the TOI and the E3 ligase enable positive cooperativity (α > 1), while negative cooperativity (α < 1) occurs when repulsive interactions inhibit the ternary complex formation. Their findings underscored the importance of positive cooperativity to obtain a productive ternary complex (Nowak et al., 2018). More specifically, the crystal structure revealed new contacts between BRD4 and VHL, as well as between the PROTAC linker and BRD4. Hence, even the productive secondary interactions yielded by the linker have a significant impact on ternary complex stabilization (Gadd et al., 2017).

To corroborate this, the same group designed a trivalent PROTAC *via* a branched linker, which showed enhanced target degradation by increasing binding valency (Imaide et al., 2020). However, recent works pointed out that positive cooperativity is not a strict requirement for efficient protein degradation (Chan et al., 2018; Zorba et al., 2018). Chan et al. described a series of VHL-based BET degraders that were active despite presenting unfavorable thermodynamics and kinetics features of ternary complex formation (Chan et al., 2018). Similarly, Gray et al. reported an example of non-cooperative binding between BRD4-dBET23-CRBN, which generated potent degraders (Nowak et al., 2018). To understand this apparent discrepancy, the formulated hypothesis is that the ternary complex formation not only depends on binding affinity rates and positive cooperativity but also on the absolute concentration of the target and the E3 ligase in cells (Guo et al., 2020). Therefore, quantitative western blots to measure their intracellular levels should be done in parallel to kinetic studies.

### The Hook Effect

**Figure 3:**
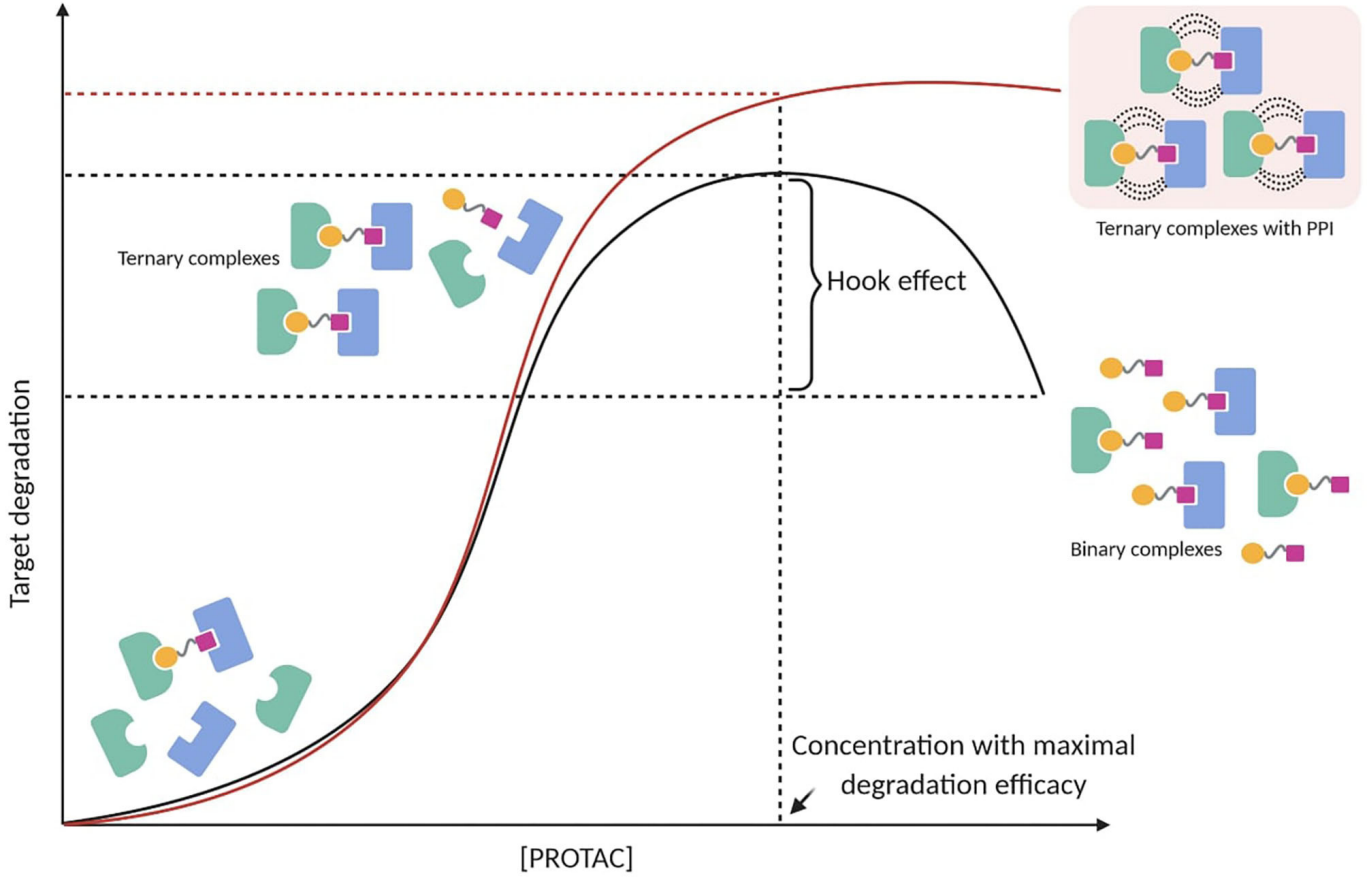
PROTAC-mediated ternary complex formation and hook effect. The hook effect is a function of PROTAC concentration (black line). A possible strategy to reduce the hook effect is increasing cooperative-binding PPIs to stabilize ternary complexes (red line).

PROTAC-mediated ternary complex hinges on established mathematical models, which illustrate the general ternary complex formation (Douglass et al., 2013; Lu and Wang, 2017). These models predict a bell-shaped dependency on PROTAC concentration, in which at high concentrations ineffective binary complexes {PROTAC-TOI or PROTAC-E3 ligase} are observed and compete with effective ternary complexes {TOI-PROTAC-E3 ligase} (Hughes and Ciulli, 2017). This well-known “hook effect” negatively impacts PROTAC's potency in a concentration-dependent manner (Figure 3). Being an intrinsic event in PROTAC pharmacology, the hook effect is difficult to avoid and often occurs at concentrations in the range of 1–10 μM, which are typically applied to test small molecules *in vitro* (Chan et al., 2018; Olson et al., 2018; Anderson et al., 2020; Shah et al., 2020; Yang et al., 2020; Zhang et al., 2020b,c; Zhou et al., 2020). Since positive cooperativity favors stable ternary complexes rather than unproductive binary complexes, it is reasonable to assume that the hook effect may be circumvented or at least reduced by improving cooperative-binding PPIs (Figure 3) (Gadd et al., 2017; Roy et al., 2017; Buhimschi et al., 2018). This has been shown in a recent study, in which a PROTAC targeting Bruton's tyrosine kinase (BTK) (MT-802) with significant positive cooperativity in the ternary complex did not present an observable hook effect below 2.5 μM (Buhimschi et al., 2018). Interestingly, locking a PROTAC in the bound conformation through macrocyclization represented an original modality to improve the cooperative binding (Testa et al., 2020). Based on their previous work (Gadd et al., 2017), Ciulli et al. hypothesized that macrocyclization would increase the energetic bias toward the productive PROTAC ternary complex rather than non-productive binary complexes. As a result, the macrocyclic PROTAC showed enhanced degradation potency and selectivity between homologous targets compared to the previous linear degrader (MZ1) (Testa et al., 2020). These findings suggest that understanding the principles of ternary complex recognition is crucial for driving the structure-based design of these chemical probes. Further structural and biophysical investigations will have to be pursued to shed light on the molecular recognition process, which has been revealed to be much more sophisticated than previously assumed.

## Covalent vs. Non-covalent Protacs

In the field, we distinguish several types of PROTACs based on the way they interact with the TOI or the E3 ligase. Covalent PROTACs contain a chemically reactive moiety able to form a covalent bond, while non-covalent ones interact through the formation of intermolecular interactions. The debate on whether it is better to use covalent or non-covalent PROTACs is on-going and here we report few studies on BTK degraders to show where this discussion stands. With the idea that preserving the sub-stoichiometric mode-of-action is important for efficient target degradation (Bondeson et al., 2015), most of the PROTACs generated so far hold non-covalent moieties, which allows the formation of a reversible and temporary-stable ternary complex. In a recent work, Tinworth et al. tried to shed light on the question mentioned above by comparing both covalent binding and reversible binding PROTACs derived from the same BTK inhibitor ibrutinib. The non-covalent IAP-based PROTAC could successfully degrade BTK, whereas a covalent version of the same PROTAC failed to induce efficient BTK degradation, despite evidence of target engagement (Tinworth et al., 2019). The authors speculated that this effect might be due to a block in ubiquitin transfer or failure in proteasome recognition, but the mechanistic insights have not yet been elucidated. Nevertheless, several examples of successful covalent PROTACs have been reported (Buckley et al., 2015; Lebraud et al., 2016; Tomoshige et al., 2016; Burslem et al., 2018). In particular, Xue et al. generated covalent irreversible BTK degraders that, in contrast to the previous findings (Tinworth et al., 2019), achieved excellent degradation potency in cells (Xue et al., 2020). Moreover, Guo et al. sought to measure the efficacy of BTK PROTACs by comparing different warhead chemistry: reversible non-covalent (RNC), reversible covalent (RC), and irreversible covalent (IRC) (Guo et al., 2020). In parallel, another extended work conducted by London's group has been pursued with the same purpose (Ronen et al., 2020). Taking advantage of cyano-acrylamide-based reversible covalent inhibitors (Serafimova et al., 2012), they both generated potent covalent BTK degraders, thereby refusing the notion that the sub-stoichiometric MoA is the only prerequisite to produce effective PROTACs. The same authors showed the importance of PROTAC intracellular accumulation and target engagement that is significantly enhanced by the cyano-acrylamide-based reversible covalent chemistry (Guo et al., 2020). To explain the efficacy of covalent PROTACs, it is postulated that their strong binding potency probably compensates for the loss of the sub-stoichiometric mode-of-action.

## How to Avoid Off-Target Effects in Protein Degradation?

PROTAC technology aims at eliminating the pool of proteins that promote pathological conditions. On the other hand, the catalytic nature of PROTAC potentially enables complete degradation of this pool which may result in toxicity issues. For instance, inhibition of BET bromodomains is tolerated, but a complete loss of BRD2 and BRD4 is lethal (Shang et al., 2009). For this reason, a sophisticated spatiotemporal control over PROTACs function would be desirable to overcome this limitation. To address this issue, PROTACs drew inspiration from conditional gene knockout (Skarnes et al., 2011), which addresses the issues related to the traditional gene knockout by eliminating specific genes at specific times. Among the plethora of endogenous activation strategies, the discovery of PhosphoPROTACs provided an effective method to couple the conditional degradation of targeted proteins to the activation state of particular kinase signaling pathways (Hines et al., 2013). PROTACs that can be conditionally activated via phosphorylation by specific growth-factor stimuli have the advantage of temporal and dosing control as well as cell-type selectivity. However, compared to endogenous activation, a more precise control may be achieved with external stimuli, as they can be precisely directed and applied to defined and localized areas of the body.

### Spatiotemporal Control Over Protein Degradation With Optogenetics and Photo-Caged Groups

Light is one of the main agents that have been used to modulate a biological response, thereby representing a suitable tool to activate or deactivate degradation with spatiotemporal precision (Mayer and Heckel, 2006). In particular, the recently emerged optogenetic tools introduced new opportunities to enable signaling regulation, including several advantages such as superior temporal and spatial resolution, easy delivery, rapid reversibility, and fewer off-target side effects (Zhang and Cui, 2015).

In the context of protein degradation, genetically encoded degrons fused to a photosensitive light–oxygen–voltage LOV2 domain allowed protein levels to be rapidly and reversibly controlled by light on a post-translational level (Bonger et al., 2014). Although this approach provided a powerful method to study biological pathways, it requires genetic manipulation, which limits its therapeutic applicability and can potentially create non-physiological protein levels within a cell.

An orthogonal approach to optogenetics is the light-induced protein degradation through photo-removable blocking groups (Hansen et al., 2015; Silva et al., 2019). Xue et al. reported a photo-caged PROTAC targeting BRD4 which displayed potent degradation activity in cells only after light irradiation (Xue et al., 2019). They used dimethoxy-2-nitrobenzyl (DMNB) group as a photolabile caging group since it can be efficiently cleaved upon irradiation at 365 nm. In parallel, Kounde et al. expanded the caged BRD4 degraders' toolbox by synthesizing potent VHL-based PROTACs (Cyrille et al., 2019).

In line with these findings, Naro et al. developed a general approach to enable light-triggered protein degradation for any small-molecule warhead. To do that, they leveraged the strategic installation of two different photocaging groups onto E3 ligase ligands recruiting VHL and CRBN (Naro et al., 2020). With the same rationale, potent Opto-PROTACs targeting dBET1 and dALK were developed as new successful chemical probes for spatiotemporal control of protein degradation (Liu and Chen, 2020). The installation of the photolabile groups was done in a position that is critical for the binding of E3 ligase, enabling its recruitment only after the caging group cleavage. These photolabile moieties were linked onto the glutarimide nitrogen in CRBN-based PROTACs, while they were installed onto the stereospecific hydroxyl group in VHL-based PROTACs (Liu and Chen, 2020). Despite the successful results, photo-caged PROTACs face limitations related to the irreversible release of active agents upon UV irradiation, which may lead to safety concerns when developing PROTAC-based therapeutics.

### Photoswitchable PROTACs

Introducing a switchable element within the chemical tool would avoid the release of active agents and allow controlled protein degradation, thereby providing a significant advantage compared to photo-caged groups. So far, the most common class of photoswitches used for the photo-control of biomolecules has been azobenzene derivatives (Beharry and Woolley, 2011; Bléger et al., 2012). The main features of these compounds include good stability, predictable geometrical changes, and facile modulation of photothermal properties (Reynders et al., 2020). Moreover, since they are relatively small, they do not significantly increase the molecular weight of the final molecule upon substitution.

The ability of azobenzenes to switch reversibly between *cis/trans* conformation upon UV light pulses grabbed the attention of PROTAC technology. Indeed, since the ratio between the two photoisomers, also known as the photostationary state (PPS), is a function of the wavelength, it can be finely regulated by specific irradiation, thereby representing an attractive system to integrate into PROTACs.

**Figure 4:**
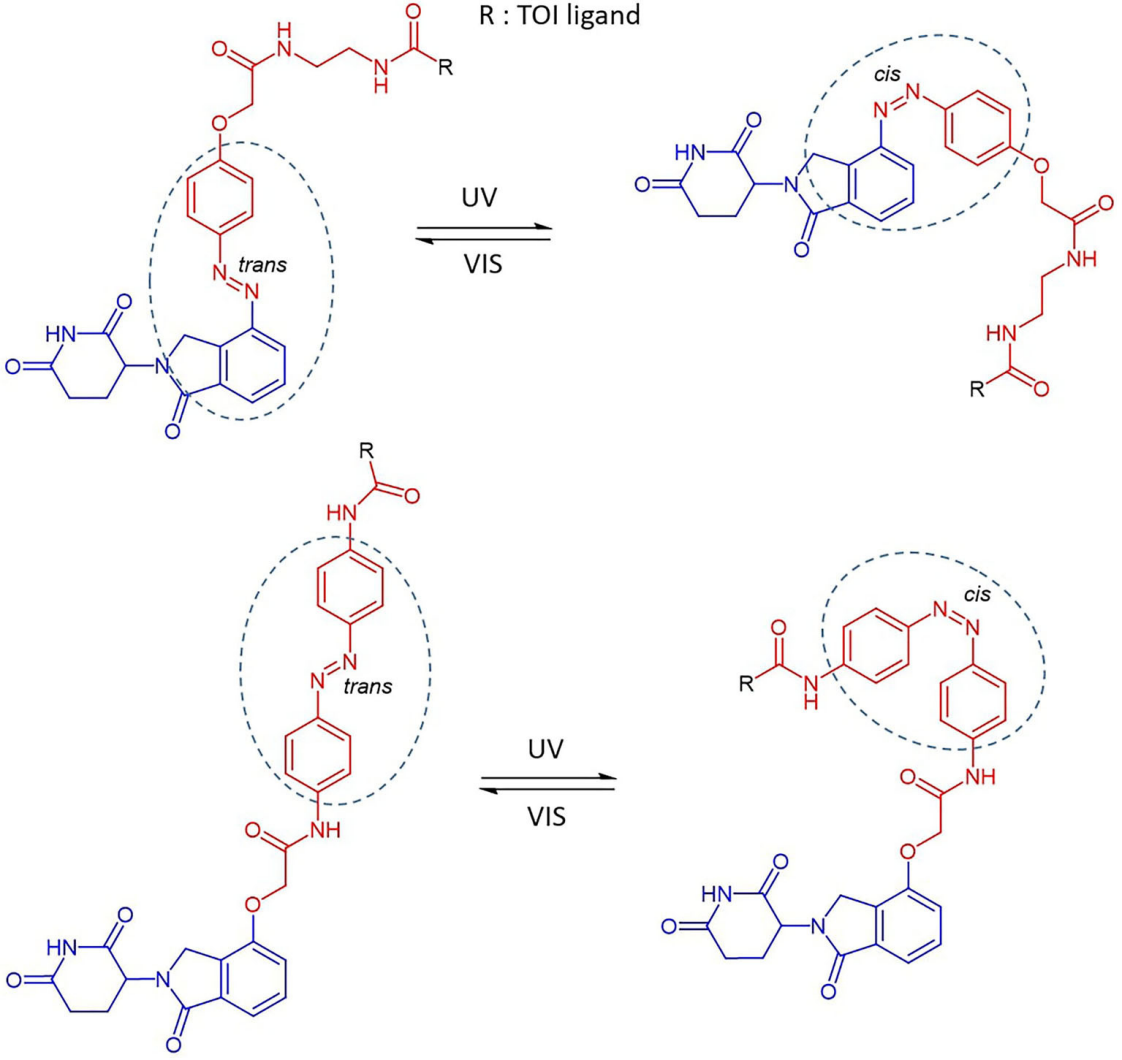
The reversible cis-trans isomerization of PHOTAC general structures by irradiation with UV-VIS wavelengths (Reynders et al., 2020). Lenalidomide (in blue), linker (in red), photoswitch groups (surrounded). The structure of the TOI ligand (R) is not shown.

Reynders et al. incorporated azobenzenes in CRBN-based PROTACs targeting either BET family proteins (BRD2,3,4) and FKBP12 (Reynders et al., 2020). The photoswitch group was introduced in one case directly on the E3 ligase recruiting moiety (lenalidomide) and, successively, in a more central position of the linker (Figure 4). With the new so-called PHOTACs in their hands, they were able to obtain potent degradation of the target upon irradiation with 390 nm light pulses, which favored the isomerization toward the active *cis* form. Reversible *cis* to *trans* isomerization could be rapidly achieved by irradiation with wavelengths >450 nm or, alternatively, through gradual thermal relaxation in the absence of light. As a result, the amount of BRD2 recovered, respectively, more quickly through irradiation with deactivating light than in cells that were left in the dark.

Using a similar strategy, Jin et al. developed CRBN-based PROTACs to target oncogenic BCR-Abl fusion and Abl proteins (Jin et al., 2020). Again, the attachment of the azo unit was done directly on the phenyl in lenalidomide. In this case, the azo-*cis* isomer was inactive, while the azo*-trans* isomer was active. The authors speculated that this is probably due to the distance defined by the linker, which in *cis* conformation is prohibitively short to allow ternary complex formation.

**Figure 5:**
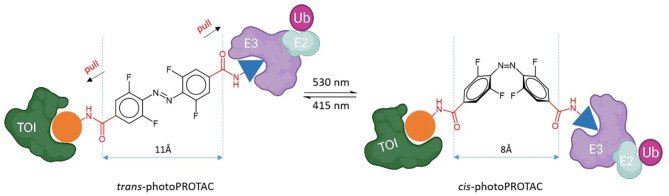
Trans-photoPROTAC and cis-photoPROTAC. trans-photoPROTAC displays an optimal distance between both warhead moieties to engage the proteins in a ternary complex; in red is shown the “pull-pull” diacid linker. cis-photoPROTAC is shorter and thus inactive (Pfaff et al., 2019).

To shed light on the impact of the *cis/trans* conformational change in protein degradation, Crew's and Carreira's labs analyzed the topological distances required to form a stable ternary complex. They observed that the critical difference in linker length between active and inactive degraders in several cases is about 3 Å, which approximately corresponds to the difference between *trans* and *cis* azobenzenes (Figure 5) (Pfaff et al., 2019). Thanks to this observation, they were able to confirm that the *azo-cis*isomer is inactive because the linker distance is too short to engage both proteins in a ternary complex. Moreover, they optimized the strategy to obtain thermally bistable photoswitches by passing from the original “push–pull” system to a “pull–pull” system (Pfaff et al., 2019). Essentially, this consisted of a reversion of the amide bond in the azobenzene linker, which led to a diacid linker bearing two electron-withdrawing substituents in *para* to the benzene moieties. As a result, the optimized photoPROTACs were thermally stable in both conformations: irradiation with 530 nm green light generated the inactive *cis*-photoPROTAC, while irradiation with 415 nm blue light led to the active *trans*-photoPROTAC (Figure 5). The possibility to avoid continued irradiation exposure represents a major improvement and highlights the relevance of bistable photoswitchable PROTACs as a promising tool for novel therapeutics.

So far, light stimuli represent the most explored external control to enable protein degradation in a spatiotemporal manner. Efficient protein degradation through *azo-cis*isomer or *azo-trans*isomer depends on precise geometrical parameters that are defined by the engagement of the E3 ligase and the TOI in a ternary complex. The common feeling is that future approaches will include photoswitchable groups with improved spectral properties, such as the shifting into the NIR/IR window for enhanced tissue penetration (Zhang et al., 2016).

## Expanding the Toolbox to Extracellular Targets Degradation

One of the key features of PROTAC technology is the possibility to target intracellular proteins. Nonetheless, there are several extracellular proteins, such as growth factors, cytokines, and chemokines, which are often responsible for aberrant signaling in multiple diseases, thereby representing attractive targets for degradation.

**Figure 6:**
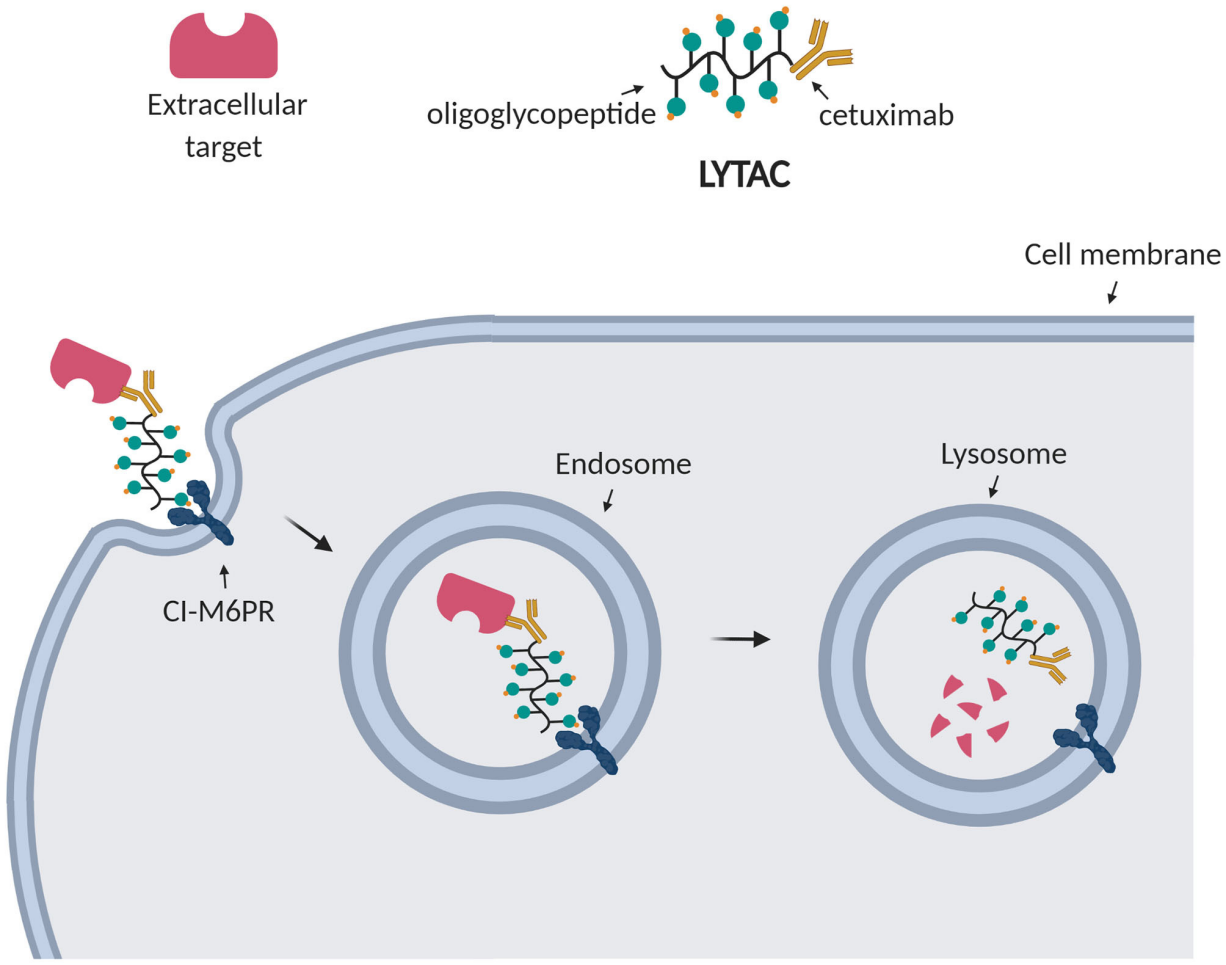
LYTAC technology. LYTACs, respectively, bind the transmembrane receptor (CI-M6PR) and the extracellular target, leading to his internalization into endosomes and final degradation into lysosomes.

For this reason, Bertozzi et al. aimed at depleting extracellular targets through lysosomal degradation using Lysosome Targeting Chimeras (LYTACs) (Banik et al., 2020). Strikingly, they enabled targeted degradation of extracellular and membrane-associated proteins by designing and synthesizing conjugates which, respectively, bind a cell surface lysosome shuttling receptor (CI-M6PR) and the extracellular domain of a target protein. Such conjugates consisted of a polypeptide with multiple ligands for CI-M6PR attached to cetuximab, an EGFR-blocking antibody (Figure 6). After treating cells with the EGFR LYTAC for 24 h, they observed a substantial decrease in the amount of EGFR compared to the control treatment. To demonstrate the widespread applicability of this platform, they successively designed LYTACs to target other membrane proteins overexpressed in cancer cells, such as CD71 and PD-L1 (Banik et al., 2020).

Their findings provided the first proof-of-concept for enhanced lysosomal degradation driven by LYTACs. The potential of this strategy resides on the new MoA to deplete extracellular and membrane-associated proteins, which is a complementary approach to proteasomal degradation by PROTACs. However, the large size of LYTACs may result in cell permeability issues.

## *In vivo* Efficacy of PROTACs

At present, PROTACs demonstrated extensive *in vitro* activity, but limited *in vivo* data are available. While the panorama of PROTAC *in vivo* studies is increasing, there is already a body of evidence showing good metabolic stability and tissue distribution properties of PROTACs (Raina et al., 2016; Ohoka et al., 2017; Saenz et al., 2017; Qin et al., 2018; He et al., 2020). Moreover, phase I clinical trials of oral administrated PROTACs (ARV-110 and ARV-471) targeting androgen and estrogen receptors are ongoing, respectively tested for prostate and breast cancer (Neklesa et al., 2017; Flanagan et al., 2019; Mullard, 2019). Preliminary data of phase I in a heavily pre-treated patient population already showed some efficacy of ARV-110 in men with metastatic castration-resistant prostate cancer (mCRPC) (2020). This trial is now expanding to phase II (2020). The chemical structure of ARV-110 is undisclosed but it is likely to have the VHL ligand incorporated (Bai et al., 2019).

Since a major limitation for *in vivo* studies is having sufficient exposure to the molecule in the relevant tissue compartment, many efforts have been done to improve the pharmacokinetic and pharmacodynamics (PK/PD) profiles of these chemical probes. This starts by implementing PK considerations into the design of PROTACs. In particular, an optimization in the linker region is likely to have a crucial impact on the ADME profile.

In a recent work, PROTAC metabolic stability has been improved by replacing the amide connectivity between the linker and CRBN recruiting moiety with an ether moiety. As expected, this modification led to improved pharmacokinetic profiles and thus good activity *in vivo* for BTK degraders (Jaime-Figueroa et al., 2020). The presence of a triazole in the linker seems to be another element to address the issues related to metabolic oxidation *in vivo* (Xia et al., 2019).

To improve tissue targeting of BET degraders with poor *in vivo* distribution, antibody conjugation has been used (Pillow et al., 2020). In this work, a VHL-based PROTAC has been attached to a CLL1-targeting antibody using a novel disulfide-based carbonate linker, thereby providing a degrader-antibody conjugate that displayed dose and antigen-dependent activity *in vivo* experiments. Impressively, a single intravenous dose of the conjugate afforded sustained *in vivo* exposures which resulted in antigen-specific tumor regressions (Pillow et al., 2020).

Besides structural modifications, other factors are responsible for driving the PK/PD relationships of PROTACs, such as protein re-synthesis rate, tissue concentration of the E3 ligase, and proteasome activity (Watt et al., 2019). As pharmacokinetics need to be taken into account from the early stage of PROTAC design, we report hereby some considerations on how PROTACs PK properties could be modulated.

## Delve Into PROTACs Cell Permeability Issues

To induce successful TOI degradation, PROTAC platforms need to simultaneously fulfill multiple requirements. Since most of the proteins-of-interest have an intracellular localization, the first step is to assure PROTAC solubility and cell permeability. Moreover, the bifunctional degrader has to prove its ability in ternary complex formation, engaging both the target and the specific E3 ligase to promote substrate polyubiquitination. Ultimately, the disease-causing protein needs to be recruited by the UPS machinery to undergo proteasomal elimination.

**Figure 7:**
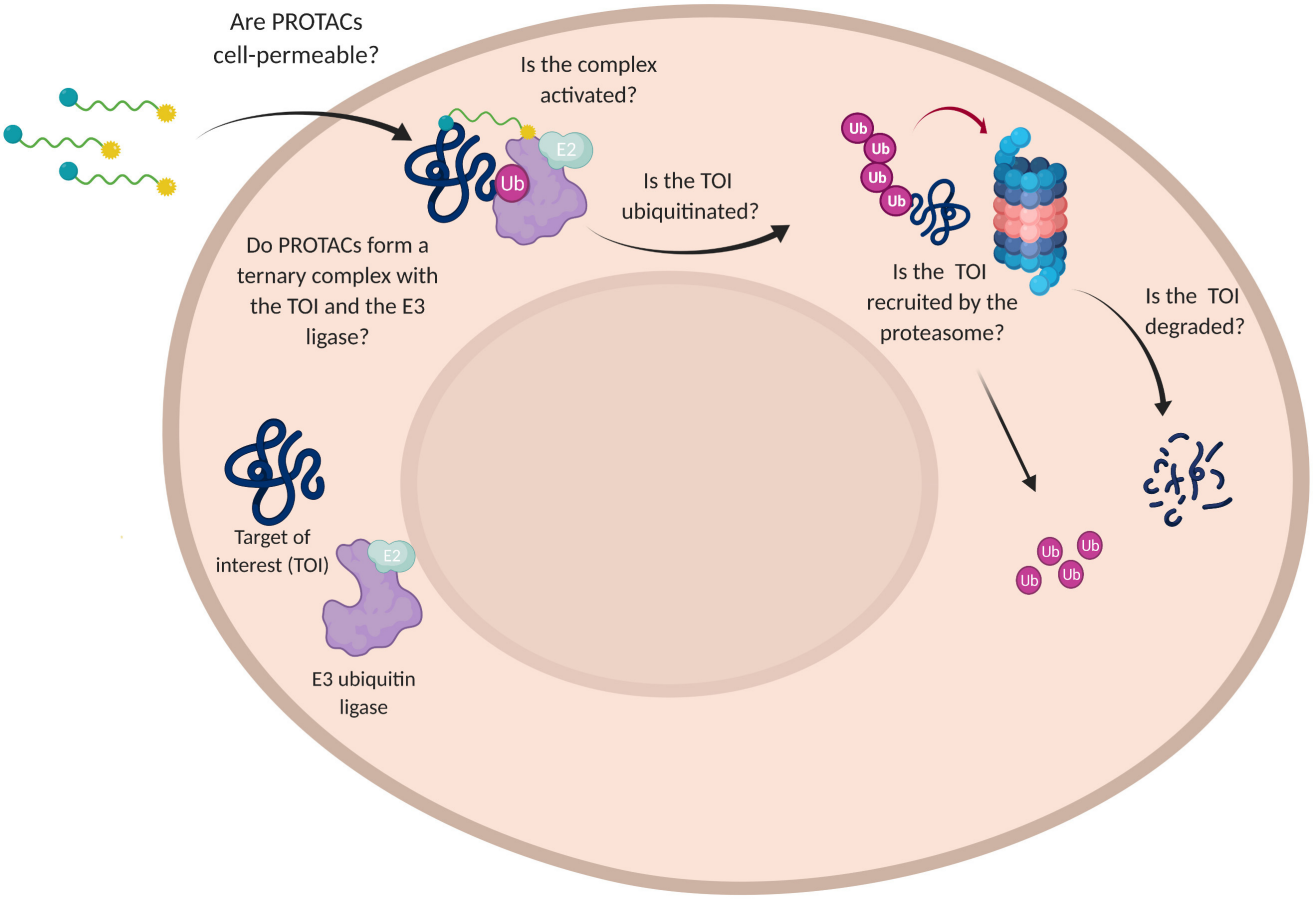
Challenges in the use of PROTAC technology. Schematic representation of the multiple questions that PROTACs need to address for affording a satisfactory TOI degradation.

In order to assess the efficacy of PROTAC candidates in TOI degradation, western blot is generally performed and target protein level evaluated (Daniels et al., 2019). However, when the desired result is not achieved, this technique is not able to detect why the degrader failed in its purpose. Did PROTAC have low solubility or was it rather unable to penetrate the cell membrane? Did PROTAC fail in boosting the formation of a stable ternary complex or TOI was not recruited by the proteasome? (Figure 7).

As discussed in the previous chapters, different methods have been developed to investigate the target engagement and the influence of the ternary complex stability on target degradation (Riching et al., 2018; Daniels et al., 2019; Roy et al., 2019); on the other hand, PROTAC cell permeability has been largely underexplored (Foley et al., 2020). Nevertheless, issues related to PROTACs permeation were known since the era of the peptide-based PROTAC technology, when Montrose and Krissansen decided to add a poly-arginine-cell penetrating peptide (CPP) to allow their bifunctional molecule to cross the cell membrane and reach the X-protein of the hepatitis B virus for degradation (Montrose and Krissansen, 2014; Zou et al., 2019). Despite the essential improvements achieved over the last few years, some PROTACs physiochemical parameters, such as high MW and a large amount of exposed polar surface areas (PSA), are unfavorable by design and represent intrinsic limitations that raise concern for their use in the clinic (Scheepstra et al., 2019).

Motivated by the lack of systematic reviews regarding PROTAC permeability issues, we intended to provide a comprehensive record of general strategies to improve passive cellular uptake and elucidate the role of active transporters, including suggestions of promising biological tools for *in vitro* evaluation of PROTACs cell penetration.

### Cell Permeability in the Chemical Space Beyond the Rule of 5 (bRo5)

With the advent of technologies such as proteomics and genomics, new therapeutic targets have been made available, including the so-called “undruggable” ones (Verdine and Walensky, 2007). The translation of these novel targets into new therapies represents the ultimate challenge that pharmaceutical R&D has to face. In particular, expanding the boundaries of oral small molecules rational design beyond Lipinski's rule of 5 (bRo5) has been suggested as one option to renovate drug discovery science (Matsson et al., 2016). In 2014, Doak et al. reported that 182 approved drugs and 303 new candidates evaluated in clinical trials had MW>500 Da. Among them, 40% of the approved drugs (*n* = 73) and 50% of clinical candidates (*n* = 153) were orally administered. The major indications accounted for these compounds included oncology, inflammation, and cardiovascular diseases (Doak et al., 2014). During the last few years, this trend seems to be confirmed by the fact that MW and lipophilicity of new drug candidates are steadily increasing, with the clear purpose of finding more suitable ligands for shaping underexplored classes of targets, such as protein-protein interactions, kinases, proteases and much more (Doak et al., 2016; Matsson et al., 2016).

Referring to their physiochemical properties, PROTACs belong to the bRo5 chemical space and share some of the common features defining this particular family of compounds, including: MW>500 Da but generally included between 700 1,000 Da since a severe drop-off in permeability is reported at MW>1,000 Da (Matsson and Kihlberg, 2017); hydrogen bond donors (HBD) >5 with few compounds exceeding 6, and hydrogen bond acceptors (HBA) >10; PSA > 200 Å (Doak et al., 2014; Matsson et al., 2016).

PROTACs are considered pharmacokinetic risky molecules mainly because increased MW is often correlated with poor solubility and decreased permeability, as well as increased active transporter-mediated efflux. Moreover, expanded lipophilicity usually reinforces overall compound permeability but at the expense of decreased solubility and increased toxic promiscuity. Michael Hann, computational and structural chemist at GSK, described this phenomenon as “molecular obesity,” stating that molecules become too big and lipophilic put at high risk their future “health” as drug candidates (Hann, 2011).

Realizing that small structural changes can greatly affect drug pharmacokinetic proprieties, it becomes crucial to delve into those features to understand how compounds in bRo5 can be adapted for overcoming size-related problems and how those strategies could be translated to the PROTAC field of study.

### Strategies to Improve PROTACs Passive Cell Penetration

Literature reports that various PROTAC chemotypes can permeate into different cell types, suggesting that simple transmembrane diffusion could be the more effective pathway for the internalization of these large-in-size compounds (Matsson and Kihlberg, 2017; Konstantinidou et al., 2019). However, the exact mechanism of PROTACs cell penetration is still partially undisclosed.

Pye et al. carried out an interesting study to evaluate the impact of molecular size and lipophilicity on the intrinsic membrane permeability for molecules bRo5. In particular, they designed a library of cyclic peptides with MW>800 Da and used the parallel artificial membrane permeability assay (PAMPA) to estimate the size-dependent permeability in this chemical space (Pye et al., 2017). It turned out that at MW>1,000 Da, molecules presenting exposed polar or charged groups had to face a sharp decrease in passive diffusion through the cell membrane. They concluded that lipophilicity needs to reside in a narrow window in order to achieve both cell permeability and aqueous solubility. At high MW this window is reduced and molecules bRo5 require a certain degree of flexibility in their chemical structure to behave in such a “chameleonic” manner (Matsson and Kihlberg, 2017).

Matsson et al. have very nicely reviewed how passive diffusion across the cell membrane can be greatly improved through different approaches aiming at reducing compounds' polarity (Matsson et al., 2016). Studies carried out on the effects of intramolecular hydrogen bonds and side chains on the pharmacokinetic of cyclic peptides have shown that bulky and lipophilic side chains can shield polar groups, like backbone amides, and ameliorate compounds permeability (Nielsen et al., 2014). Moreover, derivatization of solvent-exposed backbone amides by N-methylation has been reported in numerous works as a good strategy to improve cell permeability (Wang et al., 2014, 2015; Bockus et al., 2015). Alternatively, polarity may be reduced by designing elements able to generate intramolecular hydrogen bonds (IMHB); therefore, N-methylation or other derivatizations of heteroatoms involved in forming IMHB should be avoided (Wang et al., 2014).

One of the suggested solutions to maintain the balance between permeability, lipophilicity and solubility is to introduce conformational flexibility in the chemical structure of compounds, allowing the formation of reversible and dynamic IMHB in an environment-dependent manner (Alex et al., 2011; Matsson et al., 2016; Whitty et al., 2016). Cyclosporine A is a clear example of a bRo5 molecule showing 3D conformational flexibility: according to the environment, cyclosporine is, in fact, able to shift from a membranophilic conformation due to shielding of polarity by lipophilic side chains, to a more polar and soluble configuration, driven by the formation of reversible IMHB (Alex et al., 2011). This trend seems to be confirmed also in the case of PROTACs characterized by long flexible linkers and displaying a high number of HBDs and HBAs disposed on both protein-binding domains. This particular arrangement of HBDs and HBAs moieties, in fact, allows the spontaneous formation of IMHBs that partially shield PROTACs polarity and improves overall permeability (Klein et al., 2020).

NMR-derived structures and temperature-dependent chemical shifts showed that even rigidification, when combined with strong IMHB and solvent shielding, can enhance the bioavailability of cyclic heptapeptides (Nielsen et al., 2014). In this sense, macrocyclization has been proposed as an elegant approach for improving cell permeability and intestinal absorption for compounds in the bRo5 chemical space (Driggers et al., 2008; Mallinson and Collins, 2012). Early this year, Testa et al. have reported the design and synthesis of the first macrocyclic PROTAC MZ1, a chimeric molecule containing a VHL ligand (VH032) and the BET inhibitor JQ1. The rationale behind this strategy was to “lock” the PROTAC conformation in the bound state, using the conformational restriction of the macroPROTAC as a “molecular glue” to enhance the formation of the ternary complex. The outcome of this experiment was a satisfying degradation potency together with the optimization of compound pharmacokinetic proprieties (Testa et al., 2020).

The fact that both flexibility and rigidification can be employed as valuable strategies to improve compounds physiochemical properties seems to suggest that having a favorable IMBH parameter prevails over the intrinsic molecular conformation in determining successful permeability and thus bioavailability.

**Figure 8:**
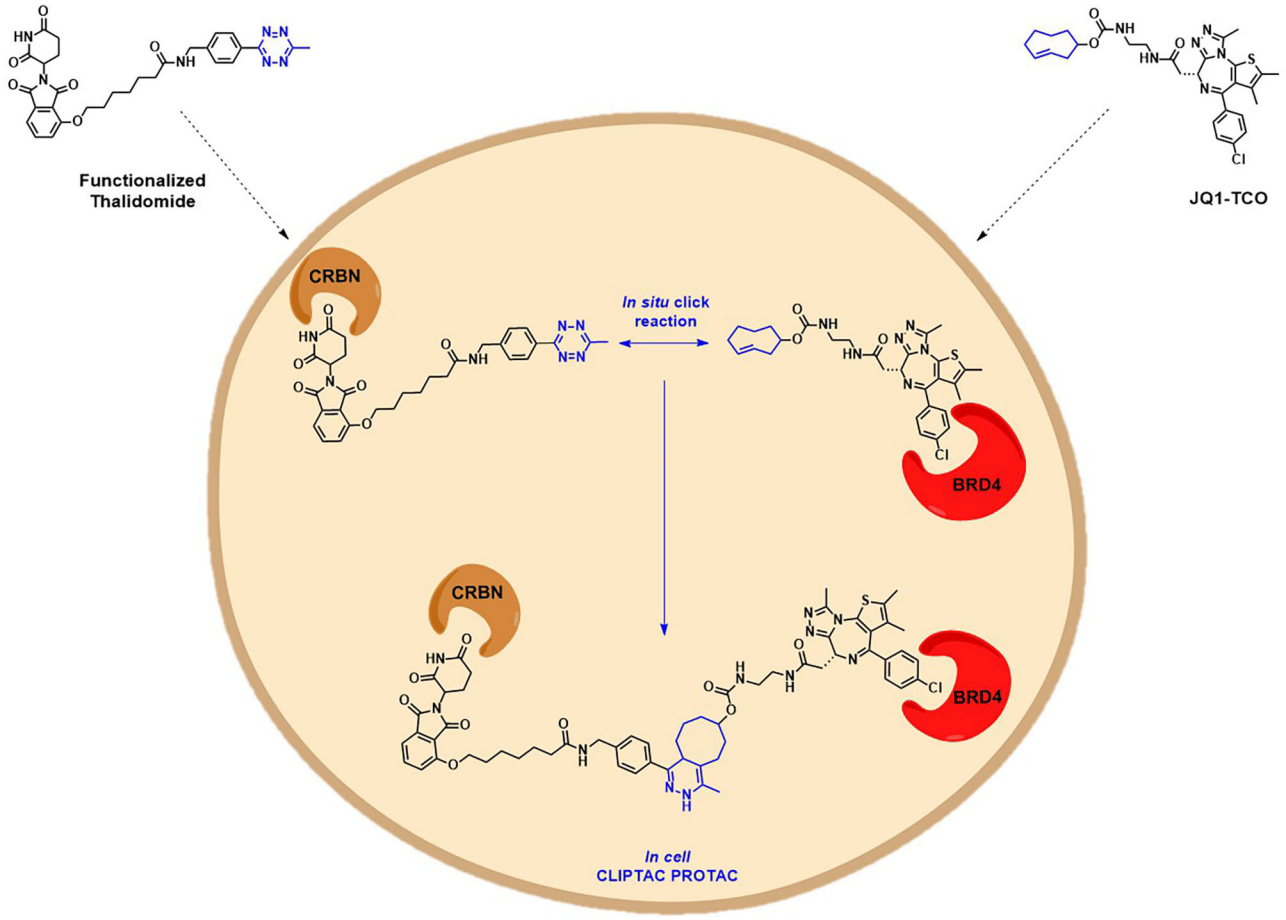
Schematic representation of the in-cell CLIPTAC PROTACs mode of action. In the example, cells are treated with TCO-tagged ligand BRD4, followed by functionalized E3 ligase binder (thalidomide). Click reaction happens *in situ* by the combination of two smaller precursors, leading to the formation of the CLIPTAC degrader.

Another interesting example addressing the permeability issue is given by Lebraud et al., who experienced a deceiving lack of TOI degradation when tried to target two oncogenic proteins, BRD4 and ERK1/2, with their pre-assembled click-formed proteolysis targeting chimeras (CLIPTACs). After the treatment of Hela cells and A375 cells, respectively, with JQ1-CLIPTAC and ERK-CLIPTAC, they perceived that the absence of activity was consistent with PROTACs lack of cell permeability. To overcome this problem, they developed an alternative chemical approach: instead of treating cells directly with high MW PROTACs, they proposed the use of the “click chemistry” to generate the bifunctional PROTACs intracellularly, by *in situ* combination of two smaller precursors that were supposed to be more permeable. The in-cell CLIPTACs demonstrated indeed an excellent degradation rate of the two key oncogenic targets (Figure 8) (Lebraud et al., 2016).

### The Role of Active Transporters for PROTACs Uptake and Efflux

It is generally accepted that passive transmembrane permeation represents the preferential route for cellular internalization of large-in-size compounds, however, the relative preponderance of passive cell permeability over transporter-mediated diffusion is affected by multiple factors and the role of transporters in PROTACs uptake is still unclear (Dobson and Kell, 2008; International Transporter et al., 2010; Di et al., 2012).

Guo et al. have serendipitously discovered that reversible covalent chemistry could be an efficient tool for fostering PROTACs' active uptake in cells. Notably, they realized that PROTAC derivatization with a cyano-acrylamide moiety can act as a molecular transport mechanism, thus improving intracellular accumulation of the bifunctional degrader. Indeed, recent works describing the covalent reversible binding that occurs between electrophilic groups and free cysteines on the cell surface, suggested that non-permeable molecules could benefit from a thio-mediated cellular uptake to enhance their transmembrane penetration (Krishnan et al., 2014; Gasparini et al., 2015; Abegg et al., 2017). In the case of Guo's PROTAC RC-1, the increased in-cell accumulation may be the outcome of a fast and reversible reaction with intracellular glutathione, serving as a trap to enhance PROTAC retention (Guo et al., 2020).

**Figure 9:**
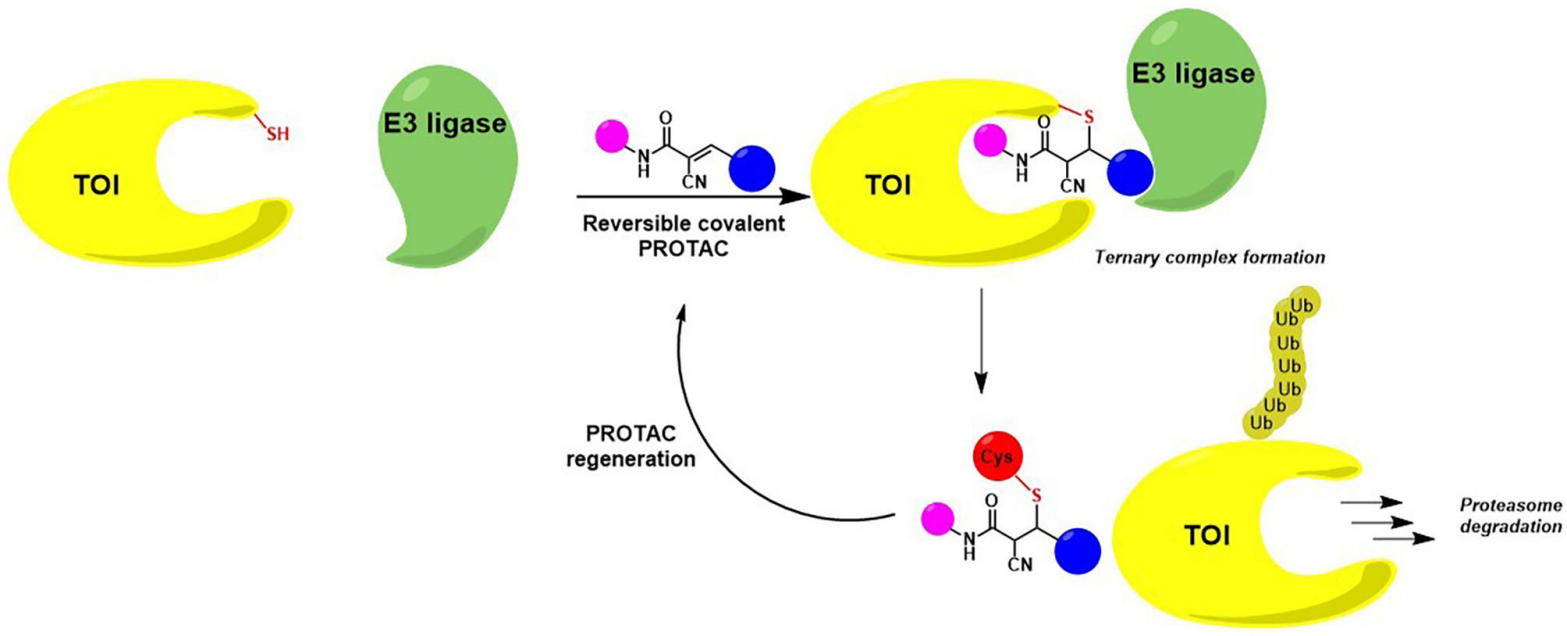
Mode of action of cyanoacrylamide-derivatized PROTAC after cellular internalization. The presence of an electrophilic group allows a reversible covalent bond between the degrader and a reactive Cys belonging to the protein of interest. TOI is eliminated while PROTAC regenerated.

Once internalized, the α-cyano-acrylamide PROTAC interacts with its putative TOI through the formation of a covalent and reversible bond, thus allowing ternary complex formation, target degradation, potent cell growth inhibition and subsequent PROTAC regeneration (Figure 9) (Guo et al., 2020).

On the other hand, it has been proven that molecules with high MW and poor passive cell permeability are more subjected to transporter-mediated efflux, whose influence increases in a size-dependent manner (Desai et al., 2013). The ATP-binding cassette (ABC) and the solute carriers (SLCs) represent two efflux transporter superfamilies that are determinant in modulating intracellular concentrations of drugs and drug-like candidates. In particular, forty-nine ABC human proteins have been identified, including the well-known P-glycoprotein (ABCB1) responsible for the ineffectiveness of numerous therapeutic compounds and multidrug resistance (International Transporter et al., 2010). Analysis of the physicochemical properties of the reported ligands for these transporters revealed significant differences between members of the ABC superfamily and the SLCs. ABC substrates present, in fact, higher lipophilicity and increased MW, thus suggesting that ABC-mediated efflux processes are more likely to affect molecules in the bRo5 chemical space (Montanari and Ecker, 2015; Nigam, 2015; Matsson et al., 2016). In support of this hypothesis, Powell et al. demonstrated that in some cell types PROTAC potency is jeopardized by P-gp expression. The cited work illustrates the synthesis of two degraders by conjugating CRBN ligand pomalidomide with, respectively, TAE684 and LDK378 (ceritinib), inhibitors of the Anaplastic Lymphoma Kinase (ALK).

Indeed, when Powell et al. assessed the anti-proliferative properties of their PROTACs TLR13-12 and TLR13-112 in neuroblastoma cell lines, they reported a deficiency in degradation due to the presence of the ABCB1 transporter. In order to confirm these results, they tested the same compounds both in low and high ABCB1 expressing cell lines, deducing that the anti-proliferative potency of PROTACs was greatly affected in those cells where the presence of the drug transporter was stronger. In particular, PROTACs activity was reduced compared to the parental inhibitors. Hence, for the first time, it was demonstrated that PROTACs could be P-gp substrates and thus be subjected to transporter-mediated efflux processes (Powell et al., 2018). It should also be noted that ALK inhibitor ceritinib, used for the conception of TLR13-112, is a well-known P-gp substrate (Katayama et al., 2016), thus suggesting that TOI binder susceptibility to ABCB1 mediated efflux could be a delicate parameter to evaluate when choosing PROTACs building blocks.

## Biological Tools for the Evaluation of PROTACs Cell Permeability

In the past few years, cell permeability has been assessed indirectly by a mere evaluation of TOI engagement after PROTACs internalization, still, some biological methods that are routinely applied to monitor transmembrane diffusion or active uptake of drug candidates and other small molecules can be adapted for testing cellular accumulation of PROTAC candidates.

The parallel artificial membrane permeability assay (PAMPA) is unanimously considered a fast and low-cost high-throughput screening tool for the evaluation of passive membrane permeability properties of drug-like molecules (Avdeef et al., 2001, 2004; Wohnsland and Faller, 2001; Sugano et al., 2002; Zhu et al., 2002; Kansy et al., 2004). The main purpose of this bio-mimetic technique is to predict drug transcellular absorption in the preliminary screening of large molecular libraries, providing useful information on ionization state, lipophilicity and solubility of tested compounds (Kansy et al., 1998, 2004).

Recently PAMPA has been combined to the lipophilic permeability efficiency (LPE) for studying cell diffusion of VHL-based PROTACs (Klein et al., 2020). This label-free approach consists of associating permeability data coming from the PAMPA assay with compounds efficiency in passively permeate the cell membrane at a given lipophilicity (Naylor et al., 2018). Indeed, LPE can predict which structural features are amenable to cause major changes in compounds permeability, such as the capacity of flexible PROTACs to adopt conformations shielding HBDs; or evaluating the permeability increment by decreasing linker length and number of HBDs/HBAs (Klein et al., 2020).

Nevertheless, the PAMPA method is not safe from heavy drawbacks that could undermine the accuracy of its predictions. Permeation of the artificial membrane is indeed highly dependent on operational pH and because compounds are often ionizable, the design of perfect experimental conditions could slow down the screening pace that represents the biggest asset for this technique to be competitive (Sugano et al., 2001). Moreover, problems occur when molecules are involved in transporters-mediated mechanisms of efflux or cellular uptake since, in this case, PAMPA needs to be coupled with additional permeability assays, like the Caco-2 monolayer, to integrate information on the passive transcellular diffusion with the absorptive and secretory components of active transport (Kansy et al., 2004).

In a recent publication, Guo et al. tried to compare the permeability of the already cited cyano-acrylamide reversible covalent PROTAC RC-1 against two other BTK PROTAC candidates, IRC-1 and RNC-2, which were showing astonishing lower efficiency in BTK degradation. Since the binding affinity alone could not explain the discrepancy of the results obtained for the three molecules, the group questioned whether the intracellular concentration of RC-1 was higher than the one of IRC-1 and RNC-1 and if this eventuality was playing a major role in target engagement (Guo et al., 2020). The first attempt was conducted using the lipid-PAMPA assay, however, since their PROTACs had extremely poor permeability properties, none of them were detected in the acceptor solution. Furthermore, the scarce recovery rates indicated that compounds were retained and stuck to the membrane, confirming that in this particular case, lipid-PAMPA was not the ideal method for assessing PROTAC intracellular concentration.

A second effort consisted of extracting PROTACs from the cell lysates to perform an LC-MS analysis for quantifying their in-cell accumulation. Even though preliminary results demonstrated a slightly higher concentration of RC-1 compared to IRC-1 and RNC-1, the lysate method raised concern on the reliability of the findings because of the difficulty in distinguishing whether detected PROTACs were intracellularly located or non-specifically trapped at the cell surface.

**Figure 10:**
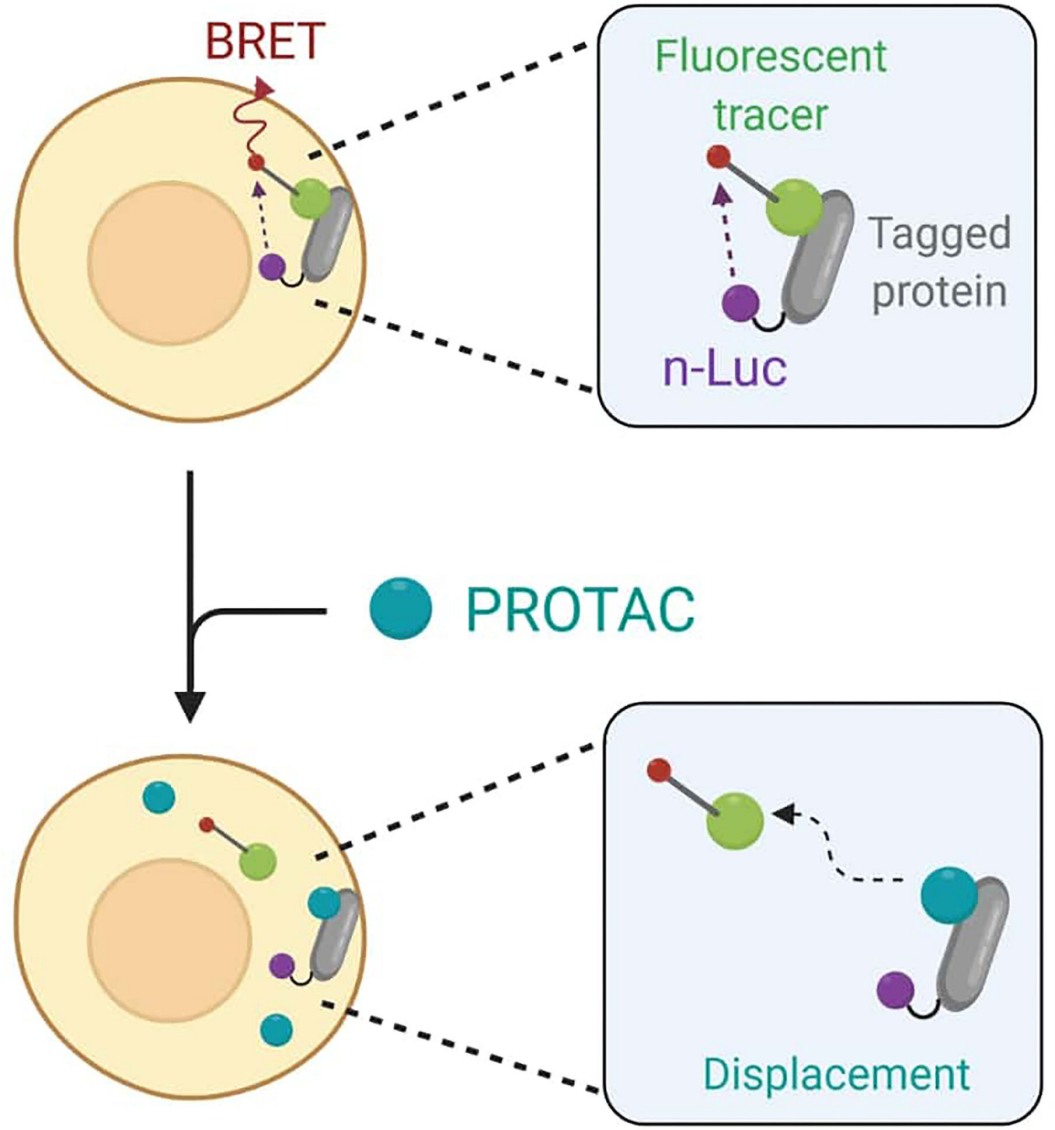
Schematic representation of the NanoBret assay. The NanoLuc (nLuc) luciferase-tagged protein interacts with a fluorescent tracer to produce a bioluminescent resonance energy transfer (BRET). The addition of PROTACs to cells results in a competition between the degrader molecule and the tracer in protein binding. The recorded BRET signal decreases accordingly to PROTAC efficiency in target engagement.

To avoid any accuracy matters, Guo et al. finally decided to employ the live cell-based NanoBRET assay that enables the quantitative determination of PROTACs intracellular concentration by assessing TOI engagement and occupancy. The NanoBRET consists of a bioluminescent resonance energy transfer (BRET) from a NanoLuc luciferase-tagged protein and a cell-permeable fluorescent tracer. In standard conditions, the tracer interacts with the NanoLuc fusion protein in cells, and BRET signal is recorded (Dale et al., 2019). However, when adding PROTACs to cells, they would compete with the fluorescent tracer for binding to the target and cause BRET signal loss as a result (Figure 10).

For BTK PROTACs, both CRBN and BTK in-cell target engagement assays were performed by transfecting HEK-293 cells, respectively, with CRBN-nLuc and BTK-nLuc fusion plasmids. Cells were then treated with CRBN and BTK tracers, followed by serial dilution with unlabeled BTK PROTACs. Recording of BRET signals confirmed that the target engagement IC_50_ values for RC-1 were 7- and 3-fold lower than the ones of RNC-1 and IRC-1. However, as Guo et al. pointed out, IC50 values can be strictly compared only under the same experimental conditions, since the tracer concentration and the expression level of NanoLuc fusion protein directly determine the unlabeled PROTAC concentration needed for obtaining the half-maximal inhibition of complex formation between these two binding partners (Robers et al., 2015).

One of the most classical approaches to determine whether a drug candidate is subjected to transporter-mediated mechanisms of efflux or cellular uptake involves the use of Caco-2 cells. The Caco-2 cell monolayer consists of a human colon adenocarcinoma cell line that is commonly used for the evaluation of drug permeability in high-throughput screening programs, since it provides a satisfying model for representing drug absorption in human jejunum (Sun et al., 2008). Moreover, the monolayer serves as a useful tool for the identification of possible substrates, activators, or inhibitors of transporters and enzymes (Lennernas, 1997). Passive transmembrane diffusion is well-characterized by the Caco-2 method, but also drugs subjected to paracellular permeation, active uptake, or ABC transporters-mediated efflux can be examined with reliable results, suggesting that the information obtained *in vitro* could be translated for *in vivo* prediction of ADME mechanisms and drug-drug interactions (Artursson and Magnusson, 1990; Troutman and Thakker, 2003; Sun et al., 2008). On the contrary, the major limitations related to this approach relay on the differences between the tight junctions and the expression of transporters and enzymes that make difficult the extrapolation of P_app_ to the actual fraction of dose absorbed (Sun et al., 2008).

For its ease of use and reliable results, the Caco-2 assay has been routinely employed in preliminary investigations of PROTACs permeability issues (Rathod et al., 2019; Atilaw et al., 2021). However, the literature equally reports that the Caco-2 test could lack sufficient sensitivity in detecting differences in cell penetration for low permeable compounds like PROTACs, thus making it impossible for these molecules to be quantitatively ranked (Foley et al., 2020).

In recent years, a HaloTag-based assay, called the chloroalkane penetration assay (CAPA), has been developed for measuring cell penetration of biomolecules in a quantitative, high-throughput, and compartment-specific manner (Peraro et al., 2018). The first protein tag (HaloTag) was designed by Wood and colleagues in 2006 and consisted of a mutant haloalkane dehalogenase, able to form irreversible and highly selective covalent bonds with synthetic ligands baring chloroalkane derivatization (Los et al., 2008).

For CAPA test, the Halo-GFP-Mito cell line is generally used to assess cell penetration for drugs with a specific cytosolic location. Indeed, this particular type of Hela cells expresses a HaloTag (GFP) merged with a mitochondria-targeting peptide (Mito) that anchors the system cytosolically oriented on the external membrane of the mitochondria (Peraro et al., 2017). Nevertheless, CAPA is not limited to the evaluation of cytosolic penetration, since using cells expressing the HaloTag fusion in other subcellular compartments like the nucleus allows permeability studies specifically in those organelles (Ballister et al., 2014; Peraro et al., 2018).

**Figure 11:**
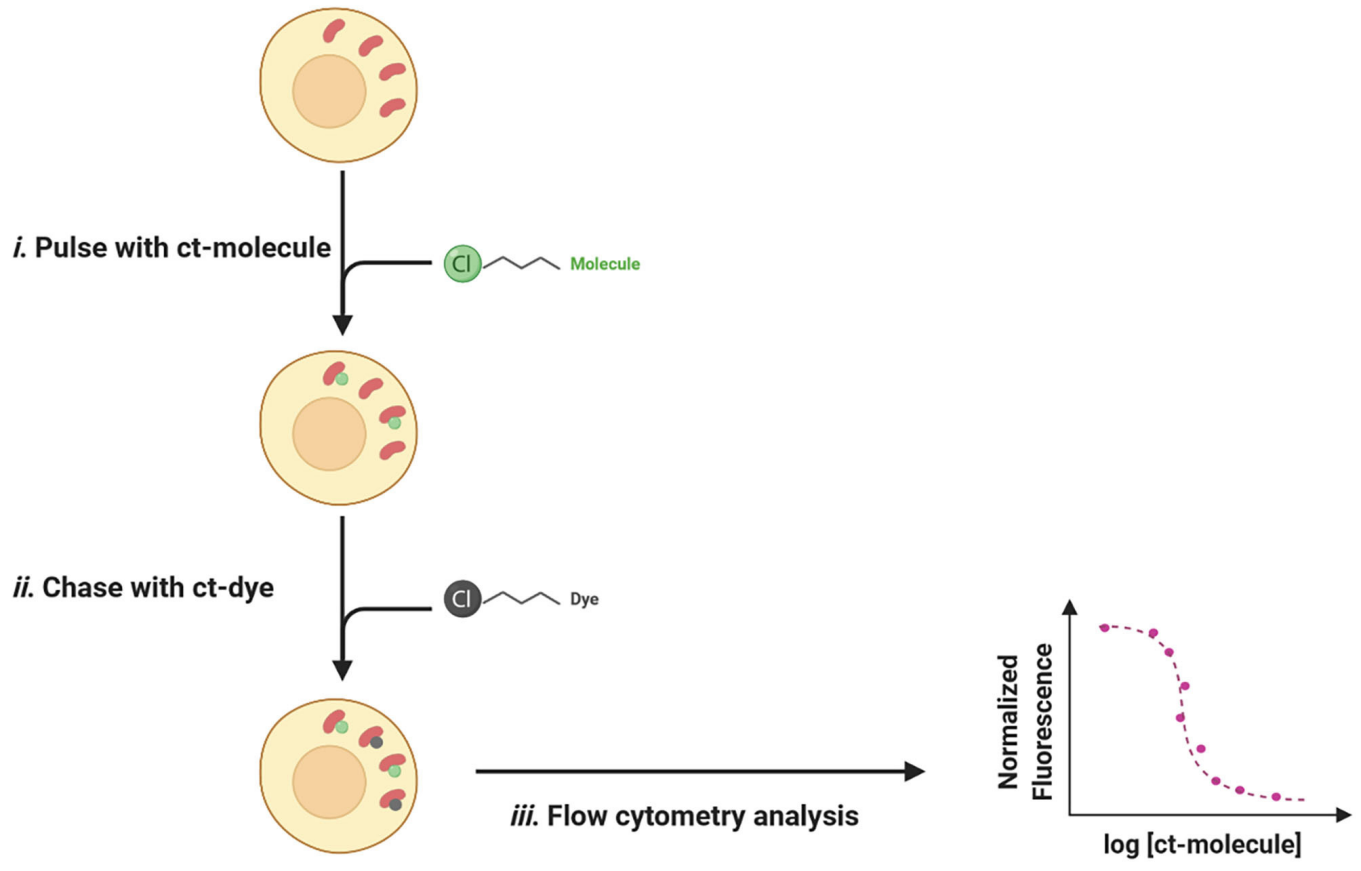
The pulse-chase procedure for the chloroalkane penetration assay (CAPA). (*i)* The Halo-GFP-Mito (red) is cytosolically oriented and bind covalently to the ct-molecules (green). (*ii)* After washing, ct-dye (gray) chases unoccupied HaloTag and provides fluorescence upon binding. (*iii)* The fluorescent signal is quantified by flow cytometry and normalized fluorescence is plotted as a function of ct-molecule concentration.

The typical setup for a CAPA experience consists of an easy pulse-chase procedure: first, cells are treated with chloroalkane-tagged molecules (ct-molecules) that, once penetrated inside the cell, will covalently bind to specific HaloTag; in a second time, cells are washed to remove unbound ligands and chased with chloroalkane-labeled dye (ct-dye). Ct-dye rapidly binds to unoccupied HaloTag and provides a fluorescent signal quantifiable by flow cytometry. The intensity of recorded fluorescence is inversely proportional to ct-molecules penetrated fraction and can be plotted as a function of drug concentration, thus revealing a dose-dependent relationship between the two values (Figure 11).

CAPA is a very flexible technology that quantifies cell penetration of small molecules while varying experimental parameters such as temperature, time of incubation, and serum content in media (Peraro et al., 2018). All these conditions result to be crucial for understanding passive cellular uptake and shape the cell penetration profile of selected drug candidates (Augustijns et al., 2000; Kosuge et al., 2008; Jiao et al., 2009; Shin et al., 2018). Since CAPA is not a label-free essay, Peraro et al. determined the eventual influence on cell penetration of the chloroalkane tag, especially in terms of linker length and position (Peraro et al., 2018). For doing so, they tested three peptides: the original stapled peptide ct-DD5o and two isomeric analogs, one with an N-terminus chloroalkane tag and the other with a C-terminus label (Peraro et al., 2017). Confronting the penetration profiles of the two isomers, they deduced that tag location was not affecting the permeability properties of ct-molecules; furthermore, the presence of chloroalkane labels was not fostering the penetration process on its own, since no differences were detected between the two isomers and the original ct-DD5o. Concerning linker length, Wood and colleagues evaluated if CAPA assay was able to appreciate the effects that PEG_2_, PEG_3_, and PEG_4_ chloroalkane tags could have had on permeation (Ohana et al., 2015). Their data were later corroborated by Peraro et al., when suggesting that, frequently, CAPA is not affected by the nature and/or length of the halo-linker, however, since some molecules could be sensitive to the influence exerted by chloroalkane tags, this has to be assessed on a case-by-case basis (Peraro et al., 2018).

In 2020, Foley et al. employed for the first time the CAPA assay to understand structure-permeability relationships in PROTAC technology (Foley et al., 2020). For their proof of concept, they decided to use the BRD4 degrader MZ1, exploiting a solvent-exposed *tert*-butyl group to chemically derivatize their PROTAC with a chloroalkane tag (ct-MZ1) without damaging the ternary complex formation. In order to determine how each PROTAC component was affecting the overall permeability, they also synthesized a series of ct-truncated compounds (ct-S-VHL, ct-PEG_3_-JQ1, ct-JQ1) and tested them in both CAPA and Caco-2 assay to compare the two systems. Foley et al. reported that, while the Caco-2 monolayer was not able to provide conclusive results, with CAPA technology it was possible to appreciate differences in permeability of the labeled compounds and quantitatively rank them, based on their cell penetration proprieties.

Encouraged by the outcome of this experience, they later decided to analyze the effects that linkers with different nature or lengths may have on PROTACs permeability. For doing so, they synthesized a new series of linkers conjugated with the VHL ligand (ct-PEG_6_-VHL, ct-PEG_2_-VHL, ct-alkyl_2_-VHL), and tested them using CAPA. When comparing ct-linkers-VHL penetration data with the ones from ct-VHL alone, Foley's group found out that ct-alkyl_2_-VHL and ct-VHL presented the same profile. This might be explained if we assume that better permeability results for VHL ligand-based PROTACs are achieved when limiting linker length and exposed polar surface areas.

In conclusion, CAPA technology represents a useful tool for obtaining structure-permeability relationships among closely related compounds, even those showing low cell penetration proprieties like PROTACs (Foley et al., 2020). However, when using this assay, some limitations need to be considered. CAPA is not label-free, thus it could be affected by artifacts such as cell penetration improvement driven by the chloroalkane chain, or potential degradation of ct-molecules causing tag release. Furthermore, if the intracellular concentration of PROTACs is subjected to transporters-mediated efflux, this input could not be taken into account (Peraro et al., 2018). That being said, the potential artifacts mentioned above could limit any tag-based technology; moreover, coupling CAPA with the Caco-2 monolayer could minimize tag-related constraints while allowing the study of active transporters' contribution to the overall *in-cell* PROTAC concentration.

## Conclusion and Outlooks

The advent of PROTAC technology to selectively degrade drivers of human diseases has opened up new promising perspectives in drug discovery, by offering possible solutions to overcome limitations related to the current drug development paradigm. The sub-stoichiometric mode-of-action, the possibility of targeting proteins previously considered “undruggable,” and the increased resilience to resistance mechanisms are some of the key features that affirm PROTACs success over small-molecule inhibitors. Despite the tremendous advancements and breakthroughs that pushed the first chimeric degraders into clinical trials, several challenges remain to be faced in PROTAC technology. Cell penetration represents one of the prime limitations when assessing the *in vitro* activity of potential PROTAC candidates, mainly ascribable to their large-in size nature and highly exposed polar surface area. With this in mind, it becomes crucial to embrace an early development strategy that aims at chemically conceiving structural changes with high impact on PROTACs pharmacokinetic proprieties such as permeability, absorption and oral bioavailability.

This review highlighted the main advantages of PROTAC technology over protein inhibition but also critically showcased its challenges. We presented some of the strategies to guide the rationale behind PROTAC design and to address issues related to low solubility and poor cell permeability. In the successful examples that we reported, each step of PROTAC development, starting from the choice of the TOI binder or the appropriate linker, took into consideration the major physicochemical parameters, later responsible for drug absorption and efficacy.

Ranging from the conception of low MW in-cell CLIPTAC PROTACs to α-cyano-acrylamide derivatization for boosting thio-mediated cellular uptake, we finally shed light on the relationship between chemical structure and permeability for this family of compounds.

Classical *in vitro* assays for the screening of small-molecule permeability skills, like the PAMPA or the Caco-2, now need to be adapted or coupled with new technologies for broadening their spectrum of application and including new therapeutic candidates in the chemical space bRo5 (Klein et al., 2020).

We can foresee that in the coming future, our deep understanding of PROTAC physiochemical properties will have a major impact on their SAR. This will surely benefit from emerging biological tools, more reliable and specific in yielding information on target engagement (Dale et al., 2019), monitoring the binding of PROTACs to separate binary complexes (Daniels et al., 2019) and quantitatively measuring cell penetration of biomolecules in a compartment-specific manner (Peraro et al., 2018).

## Author Contributions

CC and SP have contributed equally in the review conception and figures design. CC's writing focused on the general aspects of PROTAC technology, including recent advancements and challenges in the field. SP's part addressed PROTAC cell permeability issues, including strategies for improving cellular uptake and new biological tools. LS and ST participated in the review conception and they actively revised and corrected this work. All authors contributed to the article and approved the submitted version.

## Conflict of Interest

The authors declare that the research was conducted in the absence of any commercial or financial relationships that could be construed as a potential conflict of interest.
